# Gastro-Jejunostomy

**Published:** 1905-02-18

**Authors:** 


### GASTRO-JEJUNOSTOMY.

At a meeting of the South-West London Medical Society, held at the Bolingbroke Hospital on February 8th, Mr. Makins read a paper on gastro-jejunostomy.

He first described in detail the operation for anterior gastro-jejunostomy as usually performed by him. He considers it unnecessary to wash out the stomach before operation in the majority of cases, although if there is a greatly distended stomach containing a large quantity of decomposing food, it is well to attempt to improve the tone of the stomach by lavage for a few days preceding the operation. The incision Mr. Makins uses is two and a half or three inches in length just above and to the left of the umbilicus. He regards the length of the incision in the abdominal wall of importance, for, if only a short incision be made, the margins of the wound act as a sort of sphincter when the stomach and jejunum are delivered from the abdomen and help materially to shut off the area of operation from the abdominal cavity. The abdomen being opened, the peritoneum is drawn with forceps as far as is possible over the edges of the incision in the abdominal wall. This is done because the parietal peritoneum is more resistant to any chance infection than is the raw surface of the incision in the parietes. Having done this, the abdominal cavity is explored and the local conditions are carefully examined. The lower border of the stomach is drawn into the wound, and a part of its anterior wall as near as possible to the greater curvature is fixed in a Doyen's clamp. The duodeno-jejunal flexure is found, and a portion of the jejunum a few inches from the junction is similarly fixed in a Doyen's clamp. The two portions of the gut are united by a continuous posterior suture passed through the peritoneal coat of the bowel. The stomach and jejunum are then opened in the right half only of the portion of the gut which will be included in the suture arc. By this procedure a common cause of regurgitant vomiting is prevented, for when the anastomosis is completed the contents of the duodenum are prevented from entering the stomach and are directed into the jejunum. The anastomosis is completed by a continuous muco-serous suture and an anterior continuous peritoneal suture.

In the second part of his paper Mr. Makins considered the indications for gastro-enterostomy. The operation is most usually performed for some form of pyloric obstruction, and the prognosis chiefly depends upon the nature of the obstruction. In cases of pyloric carcinoma the mortality is 40 per cent., and consequently great care must be exercised in the selection of cases, and the operation ought not to be attempted in malignant obstruction when the disease is far advanced or the patient profoundly cachectic; whereas in non-malignant cases gastro-jejunostomy is one of the most successful operations in surgery. As regards congenital hypertrophic stenosis of the pylorus, Mr. Makins prefers the operation of pyloroplasty. He cautioned his hearers against operative measures in cases of hæmorrhage from gastric ulcer. The bleeding in the great majority of cases ceases spontaneously, and if operation has to be resorted to the patients are generally in so critical a state that the mortality for the operation is very high indeed.

Mr. Makins concluded by quoting illustrative cases to show the importance of first excluding any nervous cause before operating for simple dilatation of the stomach associated with constant and long-continued vomiting.

